# Applying the Hybrid Concept as a Bridge to Transplantation in Infants Without Hypoplastic Left Heart Syndrome

**DOI:** 10.1007/s00246-023-03294-8

**Published:** 2023-09-14

**Authors:** Erik L. Frandsen, Jenna S. Schauer, Brian H. Morray, David C. Mauchley, David M. McMullan, Joshua M. Friedland-Little, Mariska S. Kemna

**Affiliations:** 1 Division of Cardiology, Loma Linda University Children’s Hospital, 11234 Anderson St, Rm 4431., Loma Linda, CA 92354 USA; 2 Division of Cardiology, Seattle Children’s Hospital, Seattle, WA USA; 3 Department of Cardiac Surgery, Seattle Children’s Hospital, Seattle, WA USA

**Keywords:** Heart, Transplant, Congenital heart disease, Hybrid, Pediatric

## Abstract

Therapies to support small infants in decompensated heart failure that are failing medical management are limited. We have used the hybrid approach, classically reserved for high-risk infants with single ventricle physiology, in patients with biventricular physiology with left ventricular failure. This approach secures systemic circulation, relieves left atrial hypertension, protects the pulmonary vasculature, and allows the right ventricle to support cardiac output. This approach can be used as a bridge to transplantation in select individuals. Infants without single ventricle congenital heart disease who were treated with the hybrid approach between 2008 and 2021 were included in analysis. Eight patients were identified. At the time of hybrid procedure, the median weight was 3.2 kg (range 2.4–3.6 kg) and the median age was 18 days (range 1–153 days). Seventy five percent were mechanically ventilated and 88% were on inotropic support. The median duration from hybrid procedure to transplant was 63 days (range 4–116 days). All patients experienced a good outcome (delisted for improvement or transplanted). The hybrid procedure is an appropriate therapeutic bridge to transplantation in a carefully selected subset of critically ill infants without single ventricle congenital heart disease in whom alternate therapies may confer increased risk for morbidity and mortality.

## Introduction

Therapies to support small infants in decompensated heart failure that are failing medical management are limited. Mechanical circulatory support, which has improved transplant waitlist survival in children [1, 2], is not used as frequently in infants [3]. Outcomes for small infants who receive mechanical circulatory support have improved over time, however remain inferior for infants < 5 kg compared to larger size children, with survival in recent years around 65% for those < 5 kg [4, 5]. Our center has published experience using the hybrid stage I palliation procedure to stabilize three infants with advanced heart failure unrelated to single ventricle congenital heart disease as a bridge to transplant [6]. The hybrid procedure, which combines surgical and catheterization-based techniques, secures systemic circulation by ductal stenting, relieves left atrial hypertension and ensures adequate atrial level mixing by balloon atrial septostomy (BAS), and protects the pulmonary vasculature through bilateral pulmonary artery bands (PAB) [7]. Since the publication of our center’s initial experience, we have utilized this hybrid approach in five additional infants as a bridge to transplant. We aim to share our experience to allow identification of infants who may be supported with this approach.

## Methods

This series was compiled through retrospective review of the medical records up to latest follow-up of consecutive patients without single ventricle congenital heart disease who were treated with the hybrid approach at Seattle Children’s Hospital between December 2008 and December 2021. Institutional review board exemption was granted based on its retrospective nature.

## Results

Eight patients who underwent hybrid procedure were identified. At the time of hybrid procedure, the median weight was 3.2 kg (range 2.4–3.6 kg) and the median age was 18 days (range 1–153 days). Seventy five percent were mechanically ventilated and 88% were on inotropic support (Table 1). Pre-operative tricuspid regurgitation was moderate or greater in 38%, and pre-operative RV dysfunction was moderate or worse in 13%. Improvement in TR was seen in one patient and worsening in TR was seen in one patient after the procedure. No patient had significant worsening in RV systolic function post-operatively (Table 2). One patient was delisted due to improvement in ventricular function. All other patients survived to transplant. The median transplant waitlist duration was 48 days (range 14–135), and the median time from hybrid procedure to transplant was 63 days (range 4–116). At a median follow-up of 4.5 years (IQR 2 – 9.5 years) from transplant, all patients are currently alive and doing well (Table 3).

**Table 1:** Characteristics of patients who underwent hybrid approach as a bridge to transplant

Patient	Anatomy	Age at hybrid	Weight at hybrid	Pre-Hybrid support	Procedural detail
1	Biventricular systolic dysfunction, mild mitral valve hypoplasia (z-score -2), borderline LV hypoplasia (LVEDD z-score -1.7)	35 days	3.6 kg	Inotropes	6-mm aortopulmonary window, atrial septectomy, bilateral PAB
2	Hypertrophic cardiomyopathy, ventricular septal defect, biventricular outflow tract obstruction, Noonan’s syndrome	153 days	3.6 kg	Non-invasive positive pressure ventilation, Esmolol	4.8 mm aortopulmonary window, bilateral PAB, atrial septectomy
3	Dilated cardiomyopathy, giant LV aneurysm	10 days	3.4 kg	Mechanical ventilation, inotropes	Ductal stenting, BAS, bilateral PAB
4	Critical aortic stenosis s/p balloon valvuloplasty, severe LV dilation and dysfunction, moderate MR	5 days	2.4 kg	Mechanical ventilation, inotropes	Ductal stenting, BAS, bilateral PAB
5	Dilated cardiomyopathy, severe aortic insufficiency, severe MR	1 day	3.4 kg	Mechanical ventilation, inotropes	Aortic valve occlusion, ductal stenting, BAS, bilateral PAB
6	Dilated cardiomyopathy, severe LV dilation and dysfunction, severe MR	6 days	3 kg	Mechanical ventilation, inotropes	Ductal stenting, BAS, bilateral PAB
7	Mitral valve dysplasia with severe MR, moderate biventricular dysfunction, pulmonary hypertension	46 days	2.8 kg	Mechanical ventilation, inotropes	Ductal stenting, BAS followed by bilateral PAB 9 days later
8	Ischemic cardiomyopathy	25 days	2.7 kg	Mechanical ventilation, inotropes	BAS followed by ductal stenting and bilateral PA bands 4 days later

*LV* left ventricle, *PAB* pulmonary artery band, *BAS* balloon atrial septostomy, *MR* mitral regurgitation

**Table 2:** Pre- and postoperative echocardiogram findings in patients undergoing hybrid approach as a bridge to transplant

Patient	Preoperative TR	Preoperative RV systolic function	Preoperative PDA	Postoperative TR	Postoperative RV systolic function
1	Moderate to severe	Severely diminished	None	Moderate	Moderately to severely diminished
2	Trivial	Normal	None	Trivial	Normal
3	Trivial	Mildly diminished	Large, bidirectional shunting	Trivial	Mildly diminished
4	Moderate	Normal	Large, bidirectional shunting	Moderate to severe	Normal
5	Trivial	Mildly diminished	Large, predominately right to left shunting	Trivial	Mildly diminished
6	Mild	Normal	Large, right to left shunting	Mild to moderate	Mildly diminished
7	Mild	Mildly diminished	Moderate, bidirectional shunting	Mild	Normal
8	Moderate	Mildly diminished	Large, right to left shunting	Moderate	Mildly diminished

*TR* tricuspid valve regurgitation, *RV* right ventricle, *PDA* patent ductus arteriosus

**Table 3:** Waitlist and post-transplant outcomes in patients bridged to transplant with the hybrid approach

Patient	Age at transplant	Duration from hybrid to transplant	Support at transplant	Age at latest follow up	Alive
1	98 days	63 days	Milrinone	14 years	Yes
2	165 days	12 days	Mechanical ventilation, milrinone	11 years	Yes
3	14 days	3 days	Mechanical ventilation, milrinone, dobutamine, neuromuscular blockade	9 years	Yes
4	Delisted due to improvement. Extubated POD 5			4 years	Yes
5	117 days	116 days	Non-invasive positive pressure ventilation, milrinone	5 years	Yes
6	45 days	39 days	Mechanical ventilation, milrinone, epinephrine	2 years	Yes
7	146 days	100 days	Non-invasive positive pressure ventilation, milrinone	2 years	Yes
8	141 days	116 days	Milrinone	2 years	Yes

### Case Specifics

**Fig. 1:**
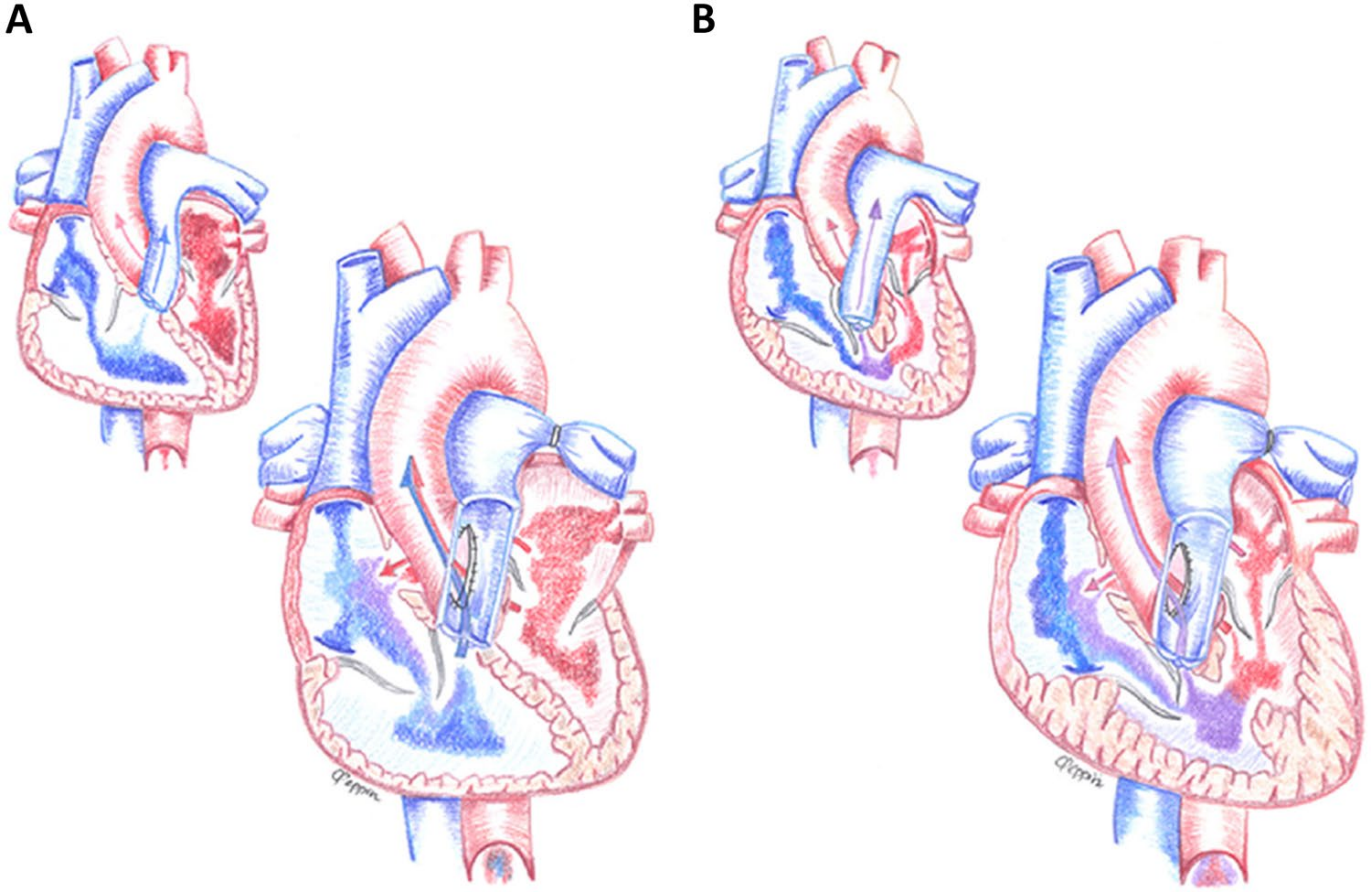
Artistic illustration of the anatomy and direction of blood flow in patient 1 (**A**) and patient 2 (**B**) before and after the hybrid procedure with creation of an aortopulmonary window. Reprinted from *The Journal of Heart and Lung Transplantation*, Vol 35, No 9, Geisser DL, et al., “Application of the Hybrid Stage 1 Palliation Concept to Patients Without Hypoplastic Left Heart Syndrome as a Bridge to Transplant”, 1133–35, Copyright Elsevier (2016)

Patient 1 was a 3.6 kg female who presented to clinic at one month of age for evaluation of a murmur. The echocardiogram was notable for small left sided structures (left ventricular end-diastolic diameter z-score − 1.7, mitral valve annulus z-score − 2, mean gradient 5 mmHg) and a severely dilated right ventricle (RV) with severe systolic dysfunction. A patent ductus arteriosus (PDA) was not present. Cardiac catheterization demonstrated left atrial (LA) hypertension worsened with atrial septal defect (ASD) occlusion (12 mmHg–28 mmHg), near systemic RV pressure, and a Qp:Qs of 2.7 due to atrial shunting. Despite inotropic support, the patient was tachypneic and tachycardic with difficulty feeding. On day of life (DOL) 35 the patient underwent surgical creation of a 6-mm aortopulmonary window, BAS and placement of bilateral PAB, allowing LA decompression and relying on the RV to augment systemic cardiac output (Fig. 1). RV systolic function did not improve post-procedure; however the patient improved clinically and was supported with milrinone and nasal cannula oxygen until transplant 2 months later. At last follow-up at 14 years of age, she is doing well on medical management for left ventricular (LV) dysfunction related to primary graft dysfunction.

Patient 2 was born at 30 weeks gestation and diagnosed with a large ventricular septal defect (VSD). At 3 months of age (3.1 kg), he was admitted with progressive heart failure symptoms and was noted to have developed severe biventricular hypertrophy with biventricular outflow tract obstruction, and a large perimembranous VSD with left to right shunting. He was listed for transplant, as myomectomy with VSD closure was felt unlikely to be successful at his size. Genetic testing revealed Noonan’s syndrome. The patient developed signs of inadequate cardiac output despite inotropic support and at 5 months of age (3.6 kg), underwent hybrid procedure with surgical creation of a 4.8 mm aortopulmonary window, bilateral PAB, and BAS (Fig. 1). After the procedure, the LV outflow tract gradient was reduced from > 100 mmHg to 27 mmHg, there was left to right shunting at the atrial level and bidirectional VSD shunting. RV outflow tract obstruction resolved. The patient remained intubated and supported on milrinone until transplant 12 days later and has done well post-transplant through 11 years of age.

Patient 3 was diagnosed at 22 weeks gestation with a large LV aneurysm. Progressive LV systolic dysfunction developed over the ensuing 6 weeks. By 28 weeks gestation, there was no antegrade aortic valve flow and severe aortic insufficiency (AI). He was carried to term and supported with mechanical ventilation and inotropes immediately after delivery. Prostaglandin E1 (PGE1) was used to maintain ductal patency to allow systemic cardiac output by the RV, as LV dysfunction was so severe that the aortic valve did not open during systole. He underwent hybrid procedure on DOL 10 with ductal stenting, bilateral PAB and BAS. He remained mechanically ventilated and on inotropes until transplant 3 days later. The ductal stent was ligated but left in place, resulting in left pulmonary artery stenosis which required arterioplasty and stent resection on postoperative day (POD) 3. He has done well post-transplant through 9 years of age.

Patient 4 was diagnosed in utero with severe LV outflow tract obstruction with LV dilation, severe systolic dysfunction and findings suggestive of endocardial fibroelastosis. Delivery was induced at 35 weeks gestation for hydrops fetalis and he was immediately intubated. PGE1 was initiated to maintain ductal patency. The initial echocardiogram showed critical aortic stenosis without insufficiency, severe LV dilation and systolic dysfunction, and moderate mitral regurgitation (MR). There was severe tricuspid valve regurgitation (TR) with normal RV function and right to left PDA flow. He underwent balloon aortic valvuloplasty on DOL 1 with reduction in the aortic valve gradient from 30 to 9 mmHg, with resultant severe AI. Hemodynamics were notable for significantly elevated LV end-diastolic pressure (20 mmHg) and LA hypertension (mean 17 mmHg). Static BAS reduced the interatrial gradient from 12 to 9 mmHg. He required initiation of inotropic support after the procedure. He ultimately underwent hybrid procedure on DOL 5. Preoperatively, there was moderate TR, which increased to severe on transesophageal echocardiogram following PAB placement with accompanying hypoxemia. Saturations improved to the mid-80% after transfusion and escalation of inotropes intraoperatively. He was extubated on POD 5. LV systolic function gradually improved over the next month while on milrinone, with improvement in AI from severe to mild. He continued to have moderate to severe tricuspid regurgitation. He was discharged at 1.5 months of age. At 2 months of age the proximal ductus was stented after a decrease in lower extremity oxygen saturation. At 8 months of age, he underwent PAB takedown with bilateral pulmonary arterioplasty, PDA ligation, Ross-Konno, aortic arch augmentation, ASD closure, and tricuspid valve repair. He was removed from the transplant list and remains well at 4 years of age.

Patient 5 presented at 21 weeks gestation with marked LV dilation and severe systolic dysfunction, severe continuous AI, severe MR, and retrograde filling of the ascending aorta by the ductus arteriosus. He was carried to term without developmental of hydrops. At birth (weight 3.4 kg), he was intubated and PGE1 started. The initial echocardiogram showed severe LV systolic dysfunction, a dysplastic aortic valve with severe AI without antegrade aortic valve flow, severe MR, and a large PDA with right to left shunting. RV function was mildly diminished and there was no TR. Shortly after birth he developed signs of myocardial ischemia with rising troponin. He underwent aortic valve occlusion with a 8 mm Amplatzer™ Vascular Plug II device, BAS, PDA stenting, and bilateral PAB. An angiogram confirmed no obstruction to coronary filling. His clinical progress was hampered by pulmonary overcirculation with low PAB gradients and increasing ASD gradient. On POD 19, cardiac catheterization revealed a 10 mmHg interatrial gradient (mean LA pressure of 19 mmHg) with low cardiac index (2.0 L/min/m2), Qp:Qs of 2:1, and mean PA pressures of 39 and 22 mmHg on the right and left, respectively. Repeat BAS was performed and an interatrial stent placed. Milrinone was maximized to reduce systemic vascular resistance therefore decreasing pulmonary blood flow. Thereafter, he tolerated enteral nutrition and was extubated to non-invasive positive pressure ventilation. He remained relatively overcirculated with oxygen saturations in the low 90% despite aggressive systemic afterload reduction but was able to wean respiratory support until transplant at 3 months of age, weight 6 kg. At transplant, aortic isthmus narrowing and right pulmonary artery narrowing at the PAB site were surgically reconstructed. He was extubated on POD 2 and discharged 4 weeks later. His post-transplant course was complicated by ectopic atrial tachycardia and moderate graft dysfunction which responded favorably to cardiac resynchronization therapy at last follow-up of 5 years of age.

**Fig. 2:**
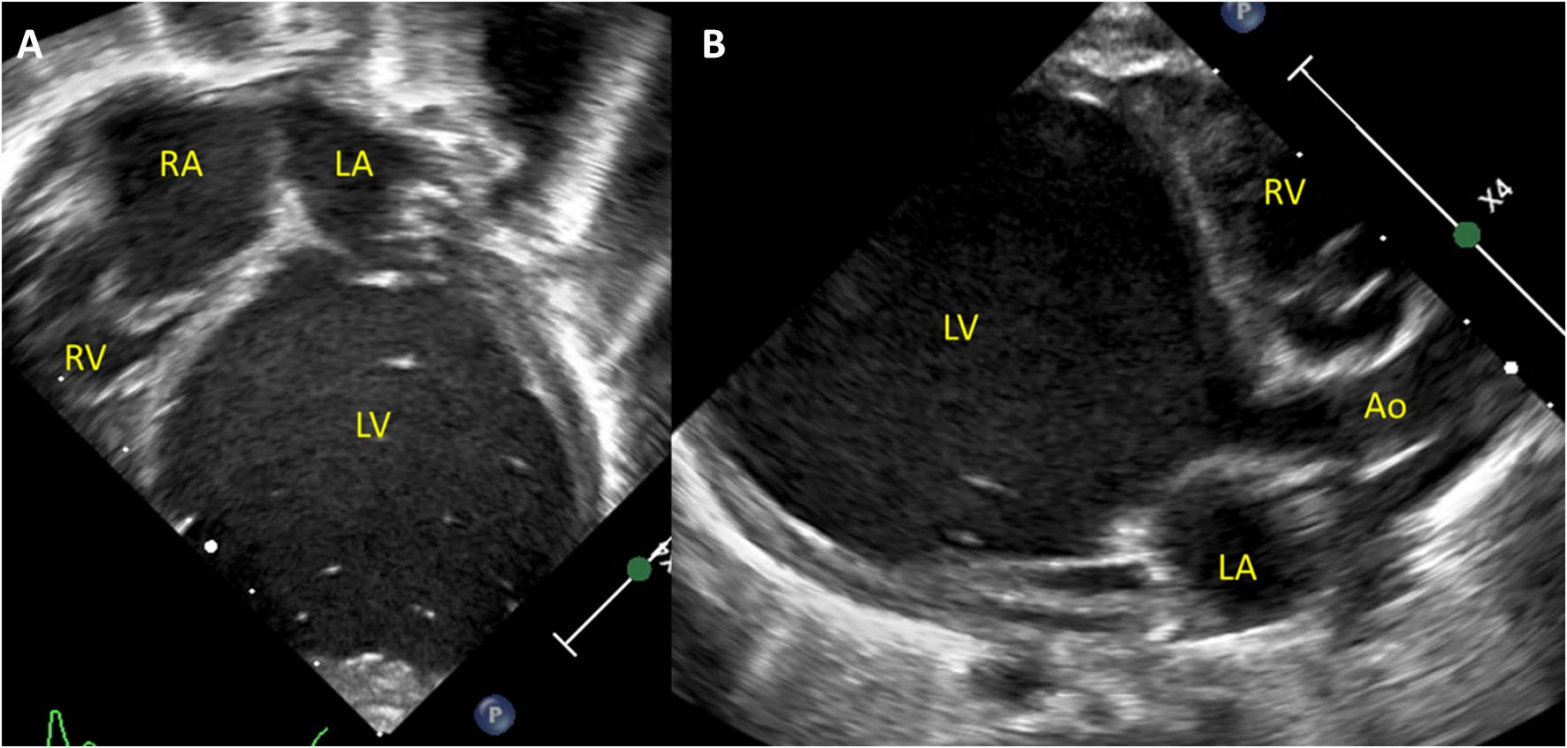
Apical 4 chamber (**A**) and parasternal long axis (**B**) echocardiogram images of patient 6 showing a severely remodeled left ventricle with severe dilation and myocardial thinning. The right ventricle is normal in size

Patient 6 was diagnosed at 33 weeks gestation with severe LV dilation and systolic dysfunction with hydrops. The patient was carried to term (birth weight 3 kg) and intubated at delivery. PGE1 and milrinone were initiated immediately. The initial echo showed severe LV dilation (z-score + 10) with severe systolic dysfunction (Fig. 2). RV systolic function was preserved and TR was mild. There was right to left PDA shunting with minimal antegrade aortic valve flow. The likelihood of myocardial recovery was felt to be minimal, so she underwent hybrid procedure with ductal stenting, PAB, and atrial septostomy. Postoperative RV function remained normal with mild TR. She failed extubation on POD 20 and had feeding intolerance requiring total parenteral nutrition. Echocardiograms showed a progressively increasing ASD gradient with persistent severe LV dysfunction and severe MR. She underwent cardiac catheterization on POD 30. Hemodynamics were notable for 4 mmHg interatrial gradient with Qp:Qs of 2:1 and CI of 3.8 L/min/m2. BAS was performed with reduction in interatrial gradient from 4 to 1 mmHg. She continued on low dose epinephrine, high dose milrinone with persistent agitation and tachycardia. She underwent transplant 7 days later, 39 days post-hybrid palliation. She was extubated on POD 4 and was discharged 3 weeks later. She remains well at 2 years old.

Patient 7 had moderately reduced biventricular systolic function on 20 week fetal echocardiogram. The patient was born at term (birth weight 3.5 kg). Immediately after birth he was supported with non-invasive positive pressure ventilation. The initial echocardiogram showed a dysplastic mitral valve with shortened chordae and moderate MR without stenosis. The LV was normal in size with moderately diminished systolic function (LV fractional shortening 23%). RV function was moderately diminished and TR was trivial. Over the subsequent 10 days, serial echocardiograms showed progressively worsening pulmonary hypertension, severe MR with LA hypertension, severe TR and persistent biventricular systolic dysfunction. Milrinone was started DOL 2. He developed renal insufficiency and was intubated on DOL 7 with improvement in vital signs and renal function. He was considered for a mitral valve repair, however, given valve morphology and his small size the likelihood of durable success was felt to be low and he was listed for transplant. The echocardiogram at 1 month of age showed a trivial PDA with bidirectional shunting, mild LV systolic dysfunction in the setting of severe MR, small ASD with left to right shunting and 15 mmHg gradient, severe LA dilation, mild TR with systemic RV pressures and mildly reduced RV systolic dysfunction. He remained ventilated on high dose milrinone. PGE1 was started to increase PDA size prior to hybrid procedure. He deteriorated shortly thereafter with post-ductal hypoxemia concerning for a pulmonary hypertensive crisis. He was taken to the catheterization lab on DOL 46. LA pressure was severely elevated at 30 mmHg with interatrial gradient of 17 mmHg, which was reduced to 6 mmHg after BAS. The transpulmonary gradient was 18 mmHg. PAB were not placed at that time due to concern for labile pulmonary vascular resistance. After BAS and PDA stenting, hemodynamics improved and supplemental oxygen need was minimal. On post-procedure day 9, he underwent bilateral PAB. He was extubated on POD 10 and tolerated enteral nutrition while supported on milrinone. He had several episodes of necrotizing enterocolitis during the 100 days waiting period from hybrid procedure to transplant, which were treated medically. His post-transplant course was uncomplicated. He was extubated on POD 2, discharged on POD 21 and remains well at 2 years old.

**Fig. 3:**
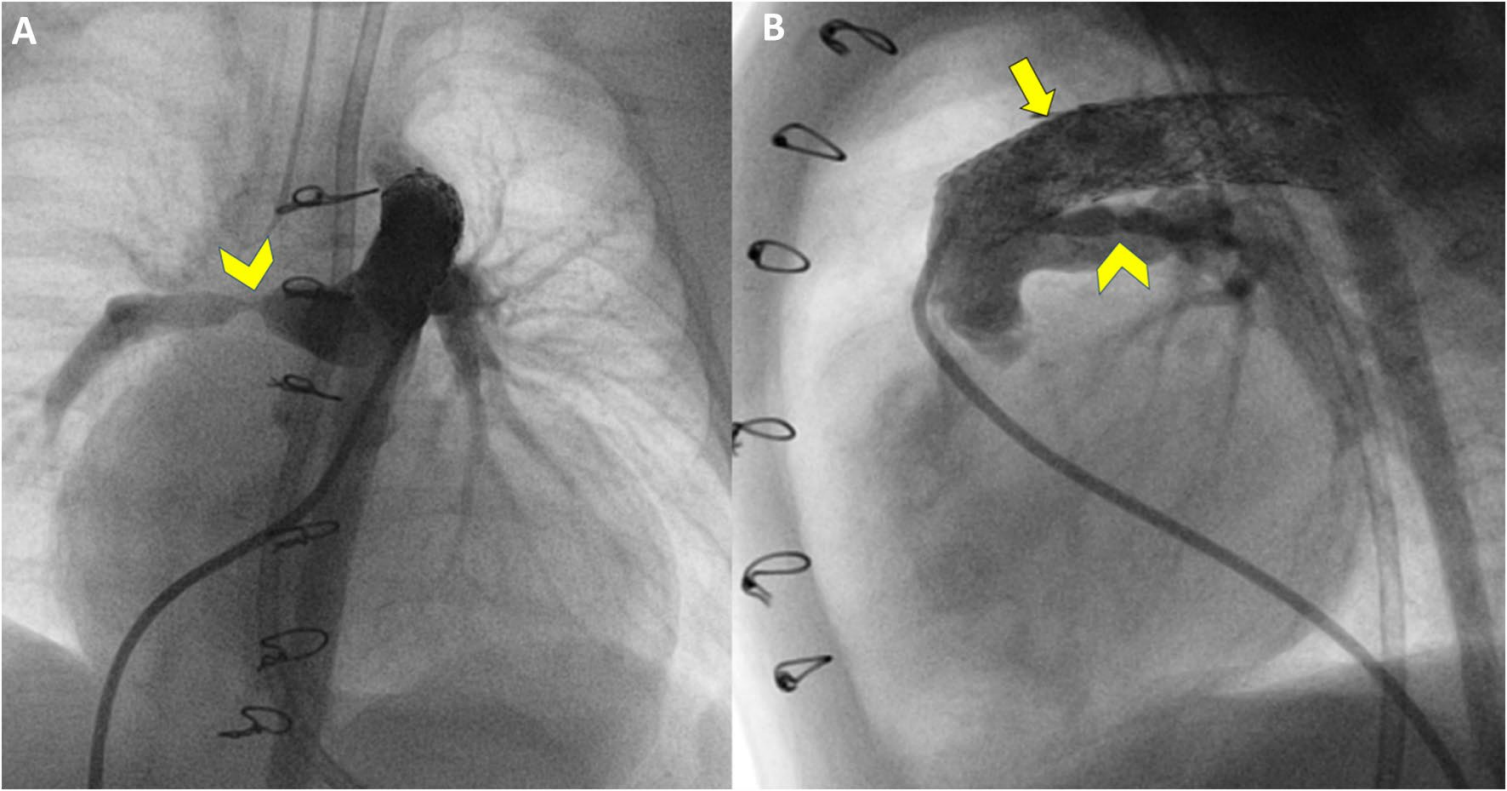
Pulmonary artery angiogram in AP (**A**) and lateral (**B**) views of patient 8 showing bilateral pulmonary artery bands (arrowheads) and ductal stent (arrow)

Patient 8 was a term 2.7 kg male who presented at birth in respiratory failure with severe LV dysfunction, preserved RV function, and moderate MR. Flow in the aortic arch was predominately from retrograde filling by the PDA and PGE1 was started. Troponin I was elevated, electrocardiogram showed ischemic changes, and coronary angiography revealed a partial filling defect in the left coronary artery extending into the left anterior descending, concerning for thrombus. A subarachnoid hemorrhage precluded thrombolytic therapy; bivalirudin was initiated to prevent further thrombus progression. On DOL 5, he underwent coronary angioplasty with improvement in left anterior descending coronary flow with minimal residual stenosis. Flow into the left circumflex remained limited, but vascular access difficulties precluded further coronary intervention. He remained intubated on dual inotropes and PGE1. Serial echocardiograms showed severe LV dysfunction, severe MR, LA hypertension with a restrictive ASD (9 mmHg gradient), and retrograde filling of the aortic arch from the PDA. The patient deteriorated on DOL 21 with pre- and post-ductal hypoxemia and lactic acidosis, which resolved immediately after BAS. A repeat echocardiogram showed persistent severe LV dysfunction with mildly reduced RV function, and moderate TR. PDA flow remained entirely right to left. On DOL 25, he underwent ductal stent and bilateral PAB (Fig. 3). He was extubated on POD 7 and was supported on milrinone and nasal cannula oxygen until transplant 4 months later. The arch was noted to be hypoplastic at transplant requiring surgical augmentation. He was extubated on POD 6, discharged on POD 26 and remains well at 2 years old.

## Discussion

The hybrid stage 1 procedure has been utilized primarily for infants with hypoplastic left heart syndrome that are at high-risk of mortality following stage 1 palliation, including infants with low birth weight, with genetic/extracardiac abnormalities, or with significant valvar or ventricular dysfunction [8, 9]. The hybrid procedure establishes a secure source of systemic blood flow, improves interatrial mixing, and protects the pulmonary vascular bed and is used as a bridge to comprehensive stage 2 palliation or cardiac transplant [10]. As presented in this case series, the concept of hybrid palliation can be applied to infants without hypoplastic left heart syndrome whose circulation can be stabilized by utilizing the RV to augment systemic cardiac output as a bridge to transplant, or as in one patient, to recovery.

The cardiac disease present in patients in this case series was heterogeneous and included primary left ventricular systolic dysfunction (patients 3, 5, 6, 8), left ventricular diastolic dysfunction (patient 1), left ventricular outflow tract obstruction (patients 2 and 4) and primary mitral valve disease (patient 7) (Table 1). The infants were critically ill, with all requiring inotropic support and all but 2 requiring mechanical ventilation. For the two patients who were older at presentation with a closed ductus arteriosus, surgical creation of an aortopulmonary window allowed the hybrid concept to be applied outside of the neonatal period.

This approach is dependent upon a right ventricle that is capable of augmenting systemic cardiac output. As such, the approach may not be as successful outside the neonatal period in the absence of a high after-load states, such as native pulmonary stenosis, pulmonary artery banding, or pulmonary hypertension, when an abrupt increase in afterload could result in right ventricular failure. The degree of RV systolic dysfunction or TR that can be tolerated following the hybrid procedure remains unclear; this is illustrated by several of our patients who had severe RV dysfunction (patient 1; in setting of significant LA hypertension with LV diastolic dysfunction) and/or significant TR (patients 1, 4, 8) but tolerated the hybrid procedure well and were successfully bridged to transplant (Table 2). We therefore remain unsure as to what degree of RV dysfunction or TR precludes a hybrid palliation. During the timeframe of this study, we have supported 4 infants with biventricular dilated cardiomyopathy to transplant without a hybrid palliation due to a combination of at least moderate RV dysfunction with at least moderate TR. In addition, longer term tolerance of hybrid physiology is less clear. Similar questions arise when considering the need for biventricular assist device support in patients with accompanying right ventricular systolic dysfunction. Often the need for right ventricular assist device support is not evident until the left ventricular assist device is in place. Importantly, the hybrid procedure does not exclude support with a ventricular assist device. Although we have not attempted it, one could conceivably use a right ventricular assist device in a single ventricle configuration [5].

The patients who underwent the hybrid procedure outside the neonatal period (patients 1, 2, 7) all had elevated RV afterload due to either RV outflow tract obstruction (patient 2) or post-capillary pulmonary hypertension (patient 1, 7). Five patients were stable enough to transfer out of the intensive care unit following recovery from their hybrid procedures and were able to rehabilitate until they received their transplant. Three patients remained mechanically ventilated until transplant, including 2 who received a transplant shortly after hybrid palliation and 1 (patient 6) who remained mechanically ventilated until transplant 39 days after hybrid. This patient did well post-transplant and was extubated on POD2 (Table 3).

Younger age and lower weight increase the risk of mortality while awaiting transplant. Thirteen percent of infants with cardiomyopathy experience waitlist mortality by 6 months after listing [11]. The need for ventilator support increases mortality risk by two-fold [12]. Therapies to support critically ill young infants listed for transplant are limited and mechanical circulatory support is used less frequently in this population due to higher morbidity and mortality in this group than in other pediatric populations [13]. The use of aggressive medical management, including mechanical ventilation, inotropic support, and neuromuscular blockade can achieve good survival to transplant, but limit the ability for rehabilitation during the waitlist period [14]. Although the use of PAB alone has been reported as a strategy in infants and young children with dilated cardiomyopathy via alteration of the ventricular-ventricular interaction [15], our described technique will relieve LV outflow tract obstruction (patients 2 and 4), unload LV volume (patients 1 and 5), or provide an immediate increase in systemic cardiac output (patients 3, 5, 6, 7, 8). When used as a surgical bridge to transplantation, the hybrid procedure may minimize exposure to human leukocyte antigen sensitization events related to blood product and homograft exposure or mechanical circulatory support [16, 17].

There are potential limitations of this approach, which should be weighed with the risks of other forms of circulatory support. Bilateral PA bands are placed at the time of the hybrid procedure to protect the pulmonary vasculature from volume overflow in the setting of an unrestrictive atrial septum and aortopulmonary shunt. As the infant grows, effective pulmonary blood flow decreases, leading to cyanosis. The degree of prograde aortic arch flow (LV systolic function) will determine preductal (cerebral) cyanosis. If the child develops unacceptable preductal cyanosis, then consideration should be given to PAB dilation, which may be met with variable success. In addition, obstruction from a protruding PDA stent may lead to a retrograde coarctation with a subsequent decrease in retrograde aortic arch flow and thus limit cerebral blood flow in those with limited prograde flow. This can be relieved by placement of a second, aortic arch stent, often positioned in a “Y” configuration through the side holes of first stent. The longer the duration from hybrid to transplant, the greater the somatic growth potential, and thus the risk of these findings. The median waitlist duration in our cohort was 48 days (range 14–135), and the median time from hybrid procedure to transplant was 63 days (range 4–116). Where waitlist times of infants exceed this, the chances of developing unacceptable cyanosis rises. In a cohort of 21 patients at our institution with HLHS bridged to transplant with the hybrid procedure where median waitlist duration was longer than the current cohort (70 days, range 2–439), 24% required catheter based interventions post-hybrid, including PAB dilation in 19% [10].

The presence of PAB and ductal stent, particularly for longer waitlist times, increases the likelihood of requiring surgical augmentation at the time of transplant. Three of the patients required augmentation of either the aorta or the pulmonary artery at transplant using donor tissue. Only one patient (patient 3) required reintervention (surgical/catheter based) in the area of PDA stent or PAB, which was due to a retained PDA stent. Although a PDA stent was utilized in those with a PDA, consideration could also be given to maintaining the infant on prostaglandin while waiting for transplant. The use of transcatheter flow restricting devices in the branch pulmonary arteries in place of external PAB, as recently described by Schranz et al., may reduce the chances of needing pulmonary arterioplasty and may allow for gradual dilation if cyanosis develops [18].

## Conclusion

The hybrid procedure is an appropriate therapeutic bridge to transplantation in a carefully selected subset of critically ill infants without single ventricle congenital heart disease in whom alternate therapies may confer increased risk for morbidity and mortality. More research is needed to identify the subset of patients most benefitted by this approach.
